# Heparinized Saline Solution vs. Saline Solution (0.9% Sodium Chloride) for the Maintenance of Dorsal Pedal Arterial Catheter Patency in Dogs Undergoing General Anesthesia: A Pilot Study

**DOI:** 10.3389/fvets.2020.00428

**Published:** 2020-07-28

**Authors:** Kazumasu Sasaki, Gonzalo Polo Paredes, Takuya Shiga

**Affiliations:** ^1^Small Animal Emergency and Critical Care Service, Sendai Animal Care and Research Center, Sendai, Japan; ^2^Akita Cerebrospinal and Cardiovascular Center, Akita, Japan; ^3^UP Anesthésie-Analgésie-Réanimation, Département des Sciences Cliniques, Ecole Nationale Vétérinaire de Toulouse, Université Toulouse-Midi-Pyrénées, Toulouse, France; ^4^Department of Anesthesiology and Perioperative Medicine, Tohoku University, Sendai, Japan

**Keywords:** arterial catheter patency, direct arterial blood pressure measurement, dog, dorsal pedal artery, heparinized saline solution, saline solution (0.9% sodium chloride)

## Abstract

Heparin is widely used as an anticoagulant solution for maintaining arterial catheter patency. In humans, increasing evidence suggests that heparinized saline solution (HS) has no advantages over a saline (0.9% sodium chloride) solution (SS) in maintaining arterial catheter patency. To date, no studies have been conducted on the effectiveness of these solutions at maintaining arterial catheter patency in veterinary medicine. The objective of this pilot study was to determine the feasibility of a study and to report the treatment efficacy comparing HS and SS for the maintenance of the dorsal pedal arterial catheter patency during direct arterial blood pressure measurements in anesthetized dogs. Client-owned dogs undergoing abdominal surgery were allocated to two groups to receive either a continuous infusion of HS or SS through the dorsal pedal artery, and the arterial pressure waveform was monitored during general anesthesia. Our feasibility outcomes included the proportion of the screened veterinary patients that completed the study and the success rate of arterial catheter placement. The clinical outcomes were assessed by the number of catheter-flushing procedures, occlusion rate, the duration of the initial catheter-flushing procedures, and the duration of catheter occlusion. Of the 51 dogs screened, 41 (80.4%) completed the study. The success rate of arterial catheter placement in the HS and SS groups were 87.5 and 80.0%, respectively. There were no differences in the number of catheter-flushing procedures and occlusion rate between groups (28.6 vs. 20.0%, relative risk [RR]: 1.429, 95% confidence interval [CI]: 0.472–4.323, *P* = 0.719 and 14.3 vs. 15.0%, RR: 0.952, 95% CI: 0.217–4.179, *P* = 1.000, respectively). No differences were found in the probability of time to the initial catheter-flushing procedure and occlusion between groups assessed by the Kaplan-Meier method (*P* = 0.546 and *P* = 0.867, respectively). This study revealed the feasibility of a study comparing HS and SS for dorsal pedal arterial catheter patency during direct arterial blood pressure measurements in anesthetized dogs. Clinical outcome analyses were underpowered and thus, could not determine the meaningful differences in treatment efficacy between the groups. However, the information gained from this study provides insight for future study designs.

## Introduction

Heparinized saline solution (HS) has been widely used as an anticoagulant to delay the formation of blood clots (1) and is empirically believed to extend the duration of arterial catheter patency during direct arterial blood pressure measurements in veterinary medicine. However, in humans, current report with respect to the use of HS for the maintenance of arterial catheter patency during direct arterial blood pressure measurements is conflicting. Several studies (2–4) and a Cochrane review (5) showed that HS did not have a significant advantage over a saline (0.9% sodium chloride) solution (SS) for maintaining arterial catheter patency during direct arterial blood pressure measurements. In contrast, Everson et al. reported that HS is more effective at preventing occlusion compared to SS (6). Furthermore, heparin exposure can result in complications such as bleeding, alternation of activated partial thromboplastin time (aPTT), and drug hypersensitivity in human patients (7, 8).

In veterinary medicine, despite the extensive use of HS for the purpose of direct arterial blood pressure measurements (9), studies to evaluate the efficacy of HS on arterial catheter patency as compared to SS have not been conducted. To the best of our knowledge, there have been no reports of complications in dogs given a continuous HS infusion during direct arterial blood pressure measurements at this stage, but the use of heparin in dogs is not risk free. Furthermore, in humans, it has been reported that SS provides acceptable direct arterial pressure waveform and is a cost-effective practice in the Intensive Care Unit (2, 3, 10, 11).

Therefore, we performed a pilot study to evaluate the feasibility of a study that compares the efficacy of HS and SS at maintaining arterial catheter patency in dogs undergoing general anesthesia. Our feasibility outcomes included the proportion of the screened veterinary patients that completed the study and the success rate of arterial catheter placement. Furthermore, we aimed to report the number of catheter-flushing procedures and occlusion rate between groups. Our hypothesis is that if a continuous infusion of SS is as effective as that of HS for the maintenance of arterial catheter patency, there would be no significant differences in the number of catheter-flushing procedures and the occlusion rate during direct arterial blood pressure measurements in dogs.

## Methods

### Design

This was a single-site, randomized, prospective, pilot study with a blinded design conducted at the Small Animal Emergency and Critical Care Service, Sendai Animal Care and Research Center. The Institutional Animal Care and Use Committee at the Akita Cerebrospinal and Cardiovascular Center approved this study (protocol number 18-02). The patient's owners gave written informed client consent for enrollment in the study.

### Veterinary Patient Enrollment

**Figure 1:**
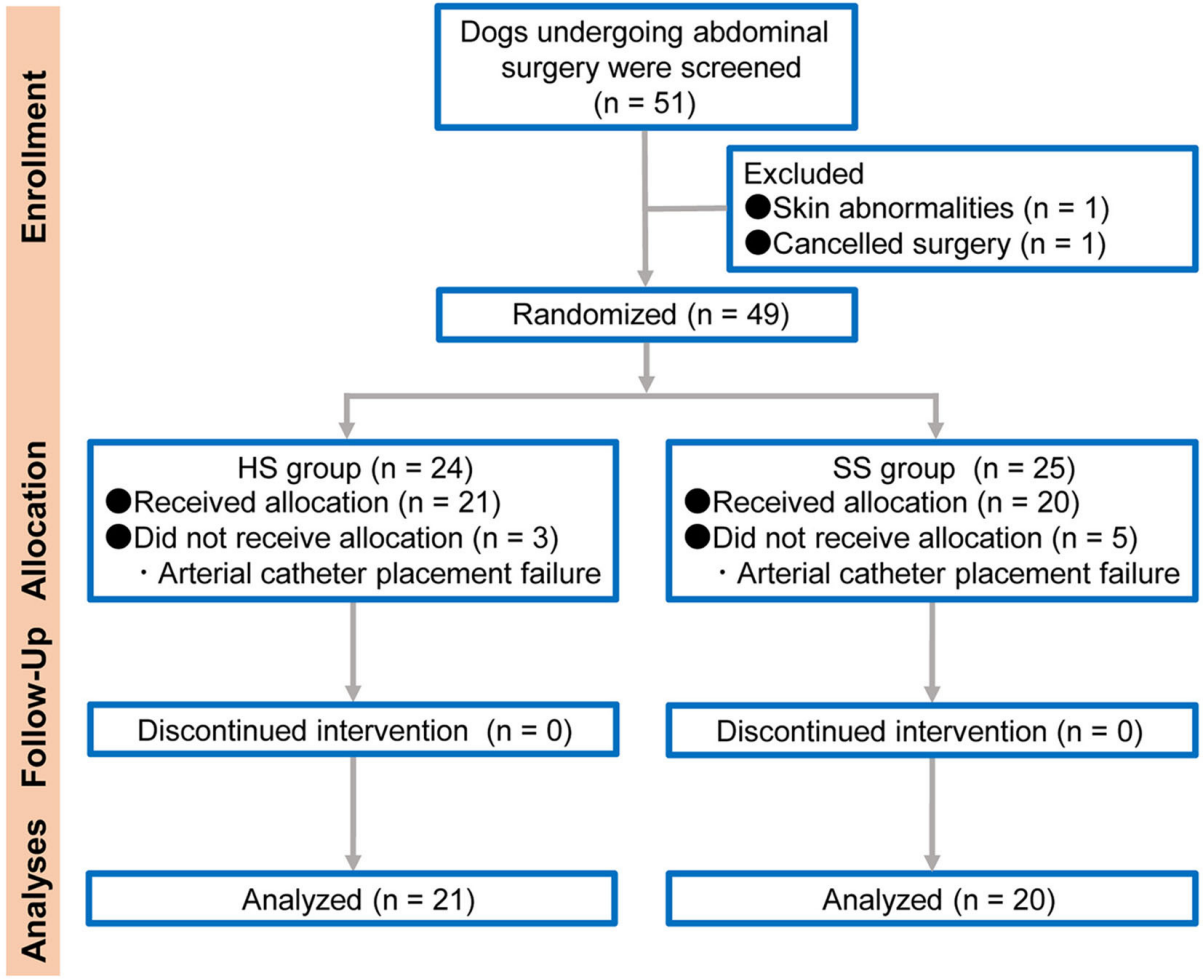
Flow diagram of veterinary patient enrollment and outcomes. HS group: the arterial catheter was maintained with continuous infusion of heparinized saline solution. SS group: the arterial catheter was maintained with continuous infusion of saline (0.9% sodium chloride) solution.

The dogs admitted to the Sendai Animal Care and Research Center for a variety of abdominal surgical procedures under general anesthesia, including ovariohysterectomy (for pyometra), gastrotomy, enterotomy, or cystolithectomy, were considered for this study. The study was performed according to the Consolidated Standards of Reporting Trials (CONSORT) statement (12) (Figure 1). Dogs aged 1 to 10 years with a body weight between 5 and 15 kg were studied. The dogs were allocated to either the HS group (the arterial catheter was maintained with continuous infusion of HS) or the SS group (the arterial catheter was maintained with continuous infusion of SS) using a web-based randomization program, RESEARCH RANDOMIZER (13). The dogs with a coagulation disorder, skin dermatitis in the arterial catheter placement site, cancellation of surgery, or arterial catheter placement failure were excluded from the study.

### Anesthesia Protocol and Patient Monitoring

The anesthetic protocol for each dog was determined in accordance with the animals' preoperative physical status including clinical symptoms, hematological examination, serum biochemical analyses, plasma electrolyte levels, coagulation system tests, echocardiography, and chest radiography. Following the induction of anesthesia, endotracheal intubation was performed in all dogs. The lungs were artificially ventilated with assist-controlled or synchronized intermittent mandatory ventilation mode using a mechanical ventilator (Pro-Next +i/+s, ACOMA Medical Industry, Tokyo, Japan) as appropriate. All dogs underwent continuous monitoring of tidal volume; respiratory rate; fraction of inspired oxygen; end-tidal sevoflurane; end-tidal partial pressure of carbon dioxide; oxygen saturation of hemoglobin; heart rate; systolic, diastolic, and mean blood pressure; and rectal temperature through a bedside multi-parameter patient monitor (Life Scope BSM-5192, Nihon Kohden, Tokyo, Japan) equipped with an automated calibration system. Arterial blood gas analyses were performed as required.

### Arterial Catheterization and Maintenance of Patency

The arterial catheters were set up as follows. Following the induction of anesthesia, the arterial catheter was placed in all dogs. The hair around the arterial catheter placement site was clipped, and the skin was prepped using chlorhexidine and 70% ethyl alcohol. We inserted a 22 gauge, 0.9 × 30 mm catheter (BD Insyte-A, Becton, Dickinson, UT, USA) into the dorsal pedal artery, and the catheter hub was connected to non-compliant pressure tubing with a disposable transducer (DX-360, Nihon Kohden, Tokyo, Japan). The transducer was connected by pressure cable into the bedside multi-parameter patient monitor (Life Scope BSM-5192, Nihon Kohden). All arterial pressure transducers were leveled and zeroed to the right atrium of the heart. The arterial catheter was continuously infused with HS, with a final concentration of 4 IU/mL (14), which was adjusted with the administration of heparin (Heparin sodium, Mochida Pharmaceutical, Saitama, Japan) to 500-mL of 0.9% sodium chloride solution or with SS (15). The veterinary technician who was not involved in the trial adjusted the study solution. The HS and SS were continuously infused at a rate of 3 mL/h using a pressure bag maintained at 300 mm Hg (6, 16). At the end of the procedure, in all dogs, the arterial catheterization was discontinued before tracheal extubation.

### Outcome Measures

The feasibility outcomes included the proportion of the screened veterinary patients that completed the study (target > 70%) and the success rate of arterial catheter placement (target > 70%). In addition, we compared the treatment effects including the number of catheter-flushing procedures and occlusion rate between HS and SS groups. The clinical evaluations and outcome measures were completed as follows. All dogs were positioned in dorsal recumbency, and they were not repositioned during the study period. The arterial pressure waveform monitoring was blindly performed by an experienced single investigator (KS). All variables including the number of catheter-flushing procedures, occlusion rate, the duration of the initial catheter-flushing procedures, and the duration of catheter occlusion were also recorded by KS. During the arterial pressure measurement, 1–1.5 mL of catheter-flushing with either HS or SS was conducted every time (maximum twice per catheter) the arterial pressure waveform was deemed to be unacceptable, aiding in sufficiently maintaining a normal arterial pressure waveform, which contains the following components: upstroke of systole, peak systole, decreasing pressure during systole, dicrotic notch, and diastole (9, 17). The fast-flush test was done for assessing an acceptable arterial pressure waveform (18). Briefly, we generated the square wave using a fast-flush test and determined the acceptable arterial pressure waveform morphology based on the number of oscillations after the square wave. We regarded an acceptable waveform as 1.5–2 oscillations below or above the baseline with a clear dicrotic notch, an overdamped waveform >2 oscillations below the baseline, or an underdamped waveform <1.5 oscillations below or above the baseline (19). If an overdamped or underdamped waveform were observed, we repressurized the pressure bag to 300 mm Hg when the pressure was not appropriate, and catheter-flushing was performed to optimize the arterial pressure waveform morphology. We considered an abnormal arterial pressure waveform morphology and the inability to withdrawal 0.5 mL of blood after the catheter-flushing procedures as indicators of catheter occlusion (20). The number of puncture attempts, number of blood samplings, and the arterial catheter placement time were recorded.

### Statistical Analyses

All results are expressed as the median (25–75% interquartile range) or as absolute numbers (%) unless otherwise mentioned. Continuous variables were assessed for normal distribution using a Shapiro–Wilk test. Differences in age, body weight, prothrombin time (PT), aPTT, the number of puncture attempts, the number of blood samplings, and the length of time that the arterial catheter was in place were evaluated using the Mann–Whitney *U*-test (Table 1). Fisher's exact test was used to determine the association between the variables with respect to the number of catheter-flushing procedures and occlusion rates. In addition, relative risk (RR) was calculated for the number of catheter-flushing procedures and the occlusion rate. Durations of the initial catheter-flushing procedures and occlusion were estimated with the Kaplan–Meier method, and the comparisons were made between groups using a log-rank test. We conducted a Cox proportional hazards regression analysis, adjusted for catheter-flushing procedures as a time-dependent risk factor, to compare the duration of occlusion between groups. A value of *P* < 0.05 was considered to indicate statistical significance. We performed statistical analyses using GraphPad Prism version 6.0 (GraphPad Software Inc., La Jolla, CA, USA), SigmaPlot version 13.0 (Systat Software Inc., San Jose, CA, USA), and Microsoft Office Excel 2016.

**Table 1:** Characteristics of the dogs enrolled in the study.

	**HS group (*n* = 21)**	**SS group (*n* = 20)**	***P*-value**
Age (y)	7.1 (6.0–8.2)	7.2 (5.7–8.2)	0.772
Sex			0.306
*Male*	4 (19.0)	7 (35.0)	
*Female*	17 (81.0)	13 (65.0)	
Body weight (kg)	9.1 (6.8–12.0)	8.9 (6.9–10.1)	0.523
Baseline PT (s)	7.7 (7.5–8.0)	7.8 (7.5–8.1)	0.933
Baseline aPTT (s)	19.8 (17.3–22.7)	18.9 (17.1–21.3)	0.54
Puncture attempt (*n*)	1.0 (1.0–1.0)	1.0 (1.0–1.0)	0.668
Number of blood sampling (*n*)	0 (0–1)	0 (0–1)	0.867
Catheter placement time (min)	93.0 (88.0–111.0)	91.0 (81.8–104.0)	0.465

*Data are presented as the median (25–75% interquartile range) or number (%). HS group: the arterial catheter was maintained with continuous infusion of heparinized saline solution. SS group: the arterial catheter was maintained with continuous infusion of saline (0.9% normal saline) solution. PT: prothrombin time. aPTT, activated partial thromboplastin time. PT and aPTT were measured using a blood coagulation analyzer (COAG 2V: A&T Corporation, Kanagawa, Japan). Reference ranges of PT and aPTT are 7.1–8.4 s and 13.7–25.6 s, respectively*.

## Results

### Feasibility Outcomes

From 51 screened adult dogs, 49 dogs (13 male and 36 female) were randomly assigned to either HS (*n* = 24) and SS (*n* = 25) groups; 41 dogs ([21 in the HS group [4 male and 17 female]] and 20 in the SS group [7 male and 13 female]) with a median weight of 7.1 kg (25–75% interquartile range: 5.8–8.2 kg) completed the study. Of the screened dogs, 96.1% (49/51) were eligible and 80.4% (41/51) completed the study. One dog was excluded from randomization for not meeting the inclusion criteria and another dog due to the cancellation of surgery. Among the randomized dogs, eight dogs were unable to be catheterized and so were excluded from the study (*n* = 3 in the HS group and *n* = 5 in the SS group). The success rates of arterial catheter placement between the HS and SS groups were 87.5% (21/24) and 80.0% (20/25), respectively. The main characteristics of the included dogs are listed in Figure 1.

### Baseline Data and Characteristics

There were no differences in any of the patient backgrounds between the groups, including the PT and aPTT, the number of puncture attempts, catheter placement time, and the number of blood samplings (Table 1).

### Clinical Outcomes

**Figure 2:**
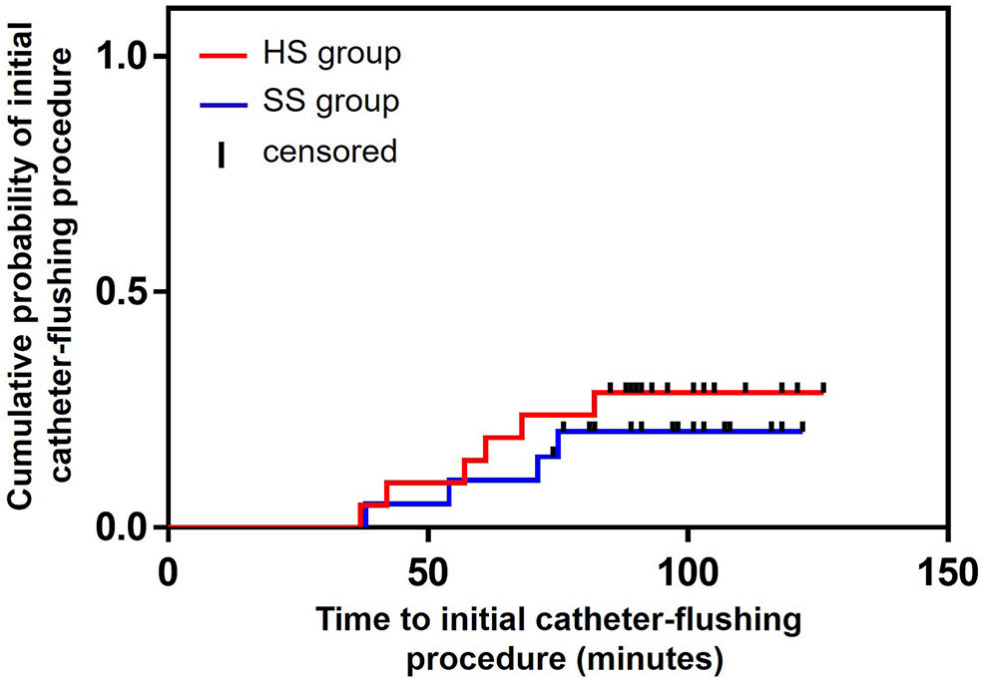
Kaplan–Meier survival curves of the probability of the duration of initial catheter-flushing procedures between the groups. HS group: the arterial catheter was maintained with continuous infusion of heparinized saline solution. SS group: the arterial catheter was maintained with continuous infusion of saline (0.9% sodium chloride) solution.

**Figure 3:**
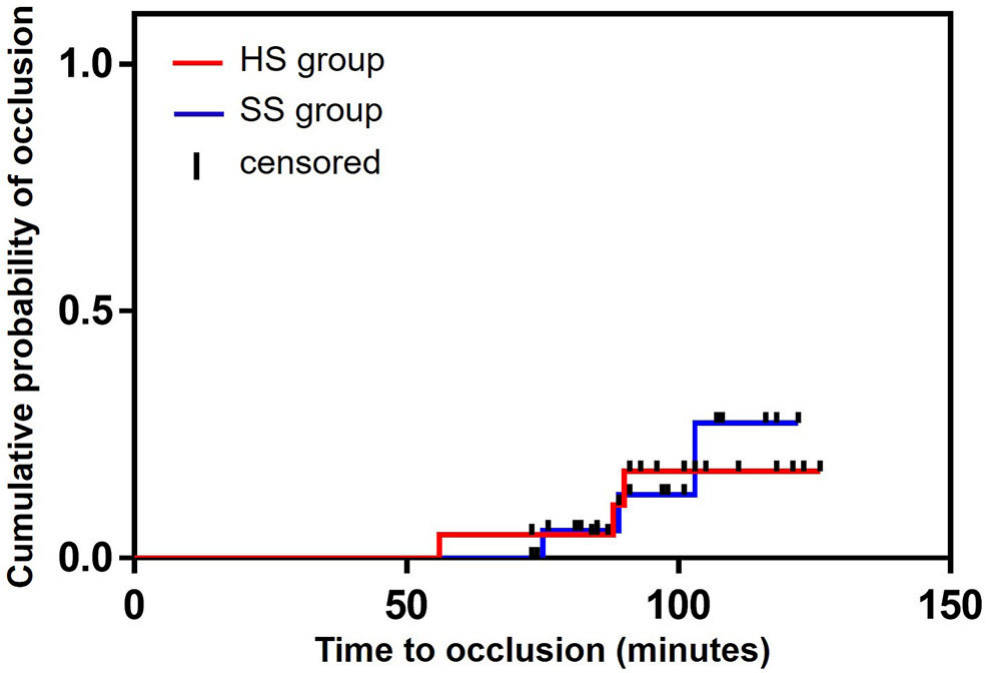
Kaplan–Meier survival curves of the probability of the occlusion between the groups. HS group: the arterial catheter was maintained with continuous infusion of heparinized saline solution. SS group: the arterial catheter was maintained with continuous infusion of saline (0.9% sodium chloride) solution.

During arterial catheter placement, there were no differences in the number of catheter-flushing procedures between the HS (6/21: 28.6%) and SS (4/20: 20.0%) groups (*P* = 0.719, relative risk [RR]: 1.429, 95% confidence interval [CI]: 0.472–4.323) (Table 2). Further, there were no significant differences in the occlusion rate between the HS (3/21: 14.3%) and SS (3/20: 15.0%) groups (*P* = 1.000, RR: 0.952, 95% CI: 0.217–4.179) (Table 2). No differences were observed between the groups with respect to the probability of the duration of the initial catheter-flushing procedures (hazard ratio [HR]: 1.472, 95% CI: 0.424–5.062, *P* = 0.546) and occlusion (HR: 0.872, 95% CI: 0.175–4.335, *P* = 0.867) analyzed by the Kaplan–Meier curves and log-rank tests (Figures 2, 3, respectively). There were no significant differences in the duration of occlusion between the HS and SS groups assessed by a Cox proportional hazards regression analysis, adjusted for catheter-flushing procedures as a time-dependent risk factor (HR: 1.325, 95% CI: 0.259–6.793, *P* = 0.736). The median (25–75% interquartile range) survival time to initial catheter-flushing procedures and catheter occlusion in the HS and SS groups was 59.0 min (45.8–66.3 min) and 57.5 min (50.0–63.5 min), and 88.0 min (72.0–89.0 min) and 89.0 min (82.0–96.0 min), respectively. Blood transfusion was not performed, and all dogs recovered from general anesthesia uneventfully. Postoperative complications included skin dermatitis, tissue ischemia, and hemostatic disorders were not observed in either group. Further, no symptoms of suspected hemostatic disorder were observed during reexamination for suture removal at 7–10 days after the removal of the arterial catheter.

**Table 2:** Frequency of catheter-flushing procedures and the rate of occlusion.

	**HS group (*n* = 21)**	**SS group (*n* = 20)**	**Relative risk**	**95% confidence interval**	***P*-value**
Catheter-flushing procedure	6/21 (28.6%)	4/20 (20.0%)	1.429	0.472–4.323	0.719
Occlusion	3/21 (14.3%)	3/20 (15.0%)	0.952	0.217–4.179	1.000

*HS group: the arterial catheter was maintained with continuous infusion of heparinized saline solution. SS group: the arterial catheter was maintained with continuous infusion of saline (0.9% normal saline) solution*.

## Discussion

### Interpretation

To the best of the author's knowledge, this is the first attempt to evaluate the feasibility of a study comparing the potential effectiveness of HS and SS at maintaining dorsal pedal arterial catheter patency in dogs undergoing general anesthesia. Among screened dogs, 80.4% (41/51) completed the study and this was an acceptable result for our criteria (target > 70%). Abdominal surgical procedures including ovariohysterectomy (for pyometra), gastrotomy, enterotomy, or cystolithectomy in dogs with a body weight between 5 and 15 kg are performed at our clinic on a daily basis. Therefore, we believe that it is feasible to conduct future studies with an adequate sample size. Although we could not compare our success rate of arterial catheter placement into the dorsal pedal artery with those of other veterinary clinicians, since no published data are available, the success rate of this study (21/24: 87.5% in HS group, 20/25: 80.0% in SS group) was acceptable based on our a priori success criteria (target > 70%). Regarding the clinical outcomes, the occlusion rate for HS (3/21: 14.3%) and SS (3/20: 15.0%) was similar and thus, our results may provide important information to guide sufficient sample number estimations to evaluate the efficacy of these solutions in future definitive studies. In addition, to estimate the treatment effects of these solutions during direct arterial blood pressure measurements in dogs, evaluating and including the concept of the minimally important difference (MID) would be advantageous for determining an appropriate sample size (21, 22). The MID is established on the basis of clinical judgment using an anchor-based and/or distribution-based approach and is used as a treatment decision threshold for the effectiveness of these solutions.

Although the sample size was insufficient to determine a meaningful result in this study, the clinical outcomes of short-term (<2 h) direct arterial blood pressure measurements were similar between the groups. In our collective clinical experiences, we have observed that long-term maintenance of dorsal pedal arterial catheter patency in dogs under sedation or when awake in the veterinary intensive care unit is challenging, as the catheters can easily become blocked. Although in humans and dogs, solid evidence has not been established that provides an ideal solution for the maintenance of arterial catheter patency at this stage, it may be worth considering the duration of the arterial catheter placement when interpreting our preliminary results. In a systematic review, Kordzadeh et al. (23) reported that in humans arterial catheter patency is feasible with both solutions if a shorter duration of arterial catheter placement within the initial time frame of 24 h is achievable, regardless of the flushing procedures (continuous infusion vs. bolus flushing), the concentration of heparin, and volume of infusion and flushing. On the other hand, once the time frame exceeds 48 h of arterial catheter placement, HS has a significant advantage with longer arterial catheter patency compared to SS (23). Alizadehasl showed that SS was as effective as HS at maintaining arterial catheter patency for 72–96 h (3). Moreover, it is not just arterial catheter patency that has been examined, a systematic review and meta-analysis reported that HS is slightly better than SS at maintaining the central venous catheter patency within a short-term period (<30 days), whereas there was not a significant difference between HS and SS in catheter patency in a long-term period (> 30 days) (24), although the intended use, the duration of catheter placement, and management method between the arterial catheter and the central venous catheter are different.

In the inclusion criteria of this pilot study, in order to minimize the confounding potential of the catheter size and the impact to the small-sized dorsal pedal arteries in dogs, we utilized only one catheter size (22 gauge). It has been reported that the ratio of the outer diameter of the arterial catheter to the vessel lumen is related to the occlusion rate in human medicine (25). Bedford showed that the incidence of radial arterial catheter occlusion was higher when using an 18 gauge catheter compared with a 20 gauge catheter, and argued that increased thrombosis may result from the greater surface area of the catheters (26). In addition, owing to the lower success rate of arterial catheterization in our clinical experiences, we excluded the dogs weighing <5 kg. Furthermore, we also excluded the dogs with a body weight >15 kg, as we routinely catheterize the dorsal pedal artery using 20-gauge catheters. We cannot discuss the impact of the catheter size on the arterial catheter patency from this study alone and thus, studies evaluating the relationship between catheter size and occlusion rate are required.

Elimination of risks of heparin-associated side effects is important for patient safety in human medicine. The guideline by Benner et al. (8) and a case study by Passannate et al. (7) sound the alarm on these heparin-related complications in human medicine. However, to the best of our knowledge, there are currently no studies reporting heparin-associated complications including bleeding, alternation of aPTT, and drug hypersensitivity in dogs given a continuous HS infusion during direct arterial blood pressure measurement. In this study, we did not encounter any clinical symptoms that were suspected to be a hemostatic disorder among the veterinary patients during their stay at our hospital and the time of reexamination for suture removal at 7–10 days after the removal of the arterial catheter. Therefore, we did not carry out postoperative hematological inspections to diagnose bleeding and altered aPTT. In addition, no clinical signs related to drug hypersensitivity were observed in any of the dogs. Thus, we could not examine the complications associated with HS in dogs from this pilot study alone, however, based on the information from human medicine, we cannot ignore the potential risks of heparin in dogs at this stage. Incorporating the planned postoperative blood collection into the evaluation item for future definitive studies would be useful for determining the risks of HS as a continuous infusion during direct arterial blood pressure measurements in dogs.

### Generalizability and Limitations

This study was conducted at a single site and thus, may not be generalizable to other veterinary facilities where the veterinary patient background and catheter size selection criteria may differ. Furthermore, there were no objective criteria to evaluate the normal arterial pressure waveform. Although the arterial pressure waveform was monitored by an experienced investigator in this study, there was a subjective side. To minimize the subjective judgment, the investigator generated square wave using a fast-flush test and by counting the number of oscillations to evaluate for the acceptable arterial pressure waveform morphology. However, it could be difficult to generalize the evaluation criteria for arterial pressure waveform between investigators.

There are several potential limitations to this veterinary clinical study. First, the impact of the diameter and length of the arterial catheter on arterial catheter patency was not evaluated. The ratio of the diameter of the catheter to the vessel lumen may influence arterial catheter patency (25). Additional studies using a smaller and/or larger size of the catheter are needed to confirm the effectiveness of maintaining arterial catheter patency by HS and SS. Second, the duration of arterial catheter placement was <2 h, and the number of blood samplings was limited. Therefore, further study is required to evaluate if similar results are observed during longer catheter placement under general anesthesia and in various clinical settings, including a veterinary intensive care unit where the dogs are treated under sedative or awake conditions. Third, previous studies indicate that the concentration of heparin does not affect the duration of arterial catheter patency within 24 h of arterial catheter placement in humans (23). Although some specific heparin concentrations are described in veterinary literature such as 1–2 IU/mL (9), 4 IU/mL (14), and 5 IU/mL (27), there is no consensus regarding the minimal heparin concentration to maintain the arterial catheter patency in dogs. In this study, the HS used had a concentration of 4 IU/mL, and this is considered much higher than that commonly used in human patients during direct arterial blood pressure measurements (2). Future studies are warranted to establish the evidence-based minimum administration dose of heparin for the maintenance of arterial catheter patency in dogs. Fourth, it has been reported in human medicine that female patients generally have smaller arteries and thus, it could result in a higher incidence of radial arterial catheter occlusion (25). Bedford indicated that the ratio of the diameter of the catheter and those of the vessel lumen are related to the generation of thrombosis on the surface of the arterial catheter (26). In the present study, patient background with respect to the allocated proportion of male and female dogs between the HS and SS groups was similar and thus, we did not perform subgroup analysis. Although information regarding sex differences in the morphology of the diameter of the dorsal pedal artery among dogs is not available, there seem to be relationships between morphological differences and occlusion rate of arterial catheters in dogs, as well as human patients. Thus, it could be worthwhile investigating the morphological differences in the dorsal pedal artery between male and female dogs, as it may be a possible factor for occlusion. Furthermore, there are currently no comparative studies with respect to the rate of dorsal pedal arterial catheter occlusion between male and female dogs during direct arterial blood pressure measurements. Therefore, future studies are required to compare the morphological characteristics and the rate of dorsal pedal arterial catheter occlusion during direct arterial blood pressure measurements between male and female dogs. Finally, we did not perform a cost analysis in this study. Cost saving is considered one of the most important benefits of switching to SS in human medicine (10, 11). There are no studies that have investigated the cost savings of switching to SS as a flush solution during direct arterial pressure measurements in veterinary clinical settings. Although, even if the cost of heparin may be insignificant in veterinary clinical settings compared with human medicine, it would be beneficial for clinical veterinarians to achieve cost-effective perioperative direct arterial blood pressure measurements. Thus, incorporating a cost analysis into the evaluation items in future definitive studies may be worth considering.

In conclusion, this pilot study provides a potential study model for comparing the dorsal pedal arterial catheter patency maintained with continuous HS and SS infusions for direct arterial blood pressure measurements using a 22 gauge catheter in dogs aged 1 to 10 years with a body weight between 5 and 15 kg undergoing general anesthesia. This study was underpowered and thus could not determine meaningful differences in the treatment efficacy between HS and SS at this stage. Future definitive studies with sufficient sample sizes are required to establish solid evidence. Further, incorporating additional evaluation items including postoperative hematological examinations and cost analyses into the design of future definitive studies would provide comprehensive information regarding this research topic.

## Data Availability Statement

The original contributions presented in the study are included in the article/supplementary material, further inquiries can be directed to the corresponding author/s.

## Ethics Statement

The animal study was reviewed and approved by The Institutional Animal Care and Use Committee at the Akita Cerebrospinal and Cardiovascular Center. Written informed consent was obtained from the owners for the participation of their animals in this study.

## Author Contributions

KS: conception and design of the study, analyzed the data, and manuscript drafting. GP: data interpretation, literature research, and manuscript preparation. TS: literature research and manuscript drafting. All authors: reviewed the manuscript and approved the final version. All authors contributed to the article and approved the submitted version.

## Conflict of Interest

The authors declare that the research was conducted in the absence of any commercial or financial relationships that could be construed as a potential conflict of interest.
